# Green Silver Nanoparticles Formed by *Phyllanthus urinaria, Pouzolzia zeylanica,* and *Scoparia dulcis* Leaf Extracts and the Antifungal Activity

**DOI:** 10.3390/nano10030542

**Published:** 2020-03-17

**Authors:** Dai Hai Nguyen, Jung Seok Lee, Ki Dong Park, Yern Chee Ching, Xuan Thi Nguyen, V.H. Giang Phan, Thai Thanh Hoang Thi

**Affiliations:** 1 Institute of Applied Materials Science, Vietnam Academy of Science and Technology, 01 TL29 District 12, Ho Chi Minh City 700000, Vietnam; nguyendaihai0511@gmail.com; 2 Graduate University of Science and Technology, Vietnam Academy of Science and Technology, Hanoi 100000, Vietnam; 3 Biomedical Engineering, Malone Engineering Center 402A, Yale University, 55 Prospect St., New Haven, CT 06511, USA; jungseok.lee@yale.edu; 4 Department of Molecular Science and Technology, Ajou University, Suwon 16499, Republic of Korea; kdp@ajou.ac.kr; 5 Department of Chemical Engineering, Faculty of Engineering, University of Malaya, Kuala Lumpur 50603, Malaysia; chingyc@um.edu.my; 6 Biomaterials and Nanotechnology Research Group, Faculty of Applied Sciences, Ton Duc Thang University, Ho Chi Minh City 700000, Vietnam; 186002007@student.tdtu.edu.vn

**Keywords:** silver nanoparticles, green synthesis, *Phyllanthus urinaria*, *Pouzolzia zeylanica*, *Scoparia dulcis*, the antifungal activity

## Abstract

Phytoconstituents presenting in herbal plant broths are the biocompatible, regenerative, and cost-effective sources that can be utilized for green synthesis of silver nanoparticles. Different plant extracts can form nanoparticles with specific sizes, shapes, and properties. In the study, we prepared silver nanoparticles (P.uri.AgNPs, P.zey.AgNPs, and S.dul.AgNPs) based on three kinds of leaf extracts (*Phyllanthus urinaria, Pouzolzia zeylanica,* and *Scoparia dulcis*, respectively) and demonstrated the antifungal capacity. The silver nanoparticles were simply formed by adding silver nitrate to leaf extracts without using any reducing agents or stabilizers. Formation and physicochemical properties of these silver nanoparticles were characterized by UV-vis, Fourier transforms infrared spectroscopy, scanning electron microscope, transmission electron microscope, and energy dispersive X-ray spectroscopy. P.uri.AgNPs were 28.3 nm and spherical. P.zey.AgNPs were 26.7 nm with hexagon or triangle morphologies. Spherical S.dul.AgNPs were formed and they were relatively smaller than others. P.uri.AgNPs, P.zey.AgNPs and S.dul.AgNPs exhibited the antifungal ability effective against *Aspergillus niger, Aspergillus flavus,* and *Fusarium oxysporum,* demonstrating their potentials as fungicides in the biomedical and agricultural applications.

## 1. Introduction

Metallic nanoparticles have attracted much attention because of their intrinsic properties that are advantageous in many applications. The nanoscale dimension guides them to penetrate easily into the hosts and cover entirely the substance surfaces. The high surface area to volume ratio [1] and the inherent characteristic of surface plasmon resonance [2] are unique. Particularly, in the life sciences, metallic nanoparticles have been rapidly developed for electronic devices, sensors, batteries, abrasive materials, semiconductor materials, catalysis, paints, detergents, adhesives, pharmaceutical products, drug delivery, and cosmetics [3,4]. Among them, silver nanoparticles (AgNPs) have the longest research history and exhibit outstanding advantages in antimicrobial applications [5]. AgNPs also inhibit formation of biofilm and antibiotic resistant strains [5]. In this antimicrobial respect, cationic polymers [6,7,8], liquid metals [9], and nitric oxide [10] also exhibited the comparable or more remarkable efficiency than AgNPs, but the complicated synthesis and high cost limited their applications within biomedical or high-tech area. Thus, AgNPs were still chosen for widely developing in all general fields. However, typical synthesis methods for AgNPs involve complicated steps and high costs, which limit their applications in biomedical and agricultural areas where large scale production of AgNPs should be considered. Typical methods to fabricate AgNPs have many drawbacks [11,12,13,14,15]. First, physical routes such as mechanical milling, laser, and thermal ablation to convert bulk materials into nanoparticles involve high energy consumption, extensively long milling/laser ablation time, and limits in modification of the formed nanoparticles (i.e., surficial functional groups and physicochemical properties) [1,11]. Second, chemical reduction routes use reducing agents (sodium borohydrate, ethylene glycol, and hydrazine hydrate) and capping agents (trisodium citrate and sodium lauryl sulphate) that are cost-inefficient, impure, toxic, and have limited reducing ability [1,4,12]. Moreover, capping agents using synthetic polymers have been become unfavorable, due to cumulative risks in humans, leading to create anti-polymer antibodies, further causing accelerated blood clearance of some pharmaceutical products [16]. These methods face further limitations when large scale synthesis is necessitated [17].

For these reasons, green synthesis of nanoparticles has been emerging as an alternative route to traditional ones. Various green sources, including microorganisms, virus, naturally reducing polysaccharides, polyols, and plant extracts were reported [4,12,18]. Microorganisms (yeasts, bacteria, fungi, and actinomycetes) may intracellularly or extracellularly synthesize metallic nanoparticles [12,18], but the disadvantages missing knowledge related to mechanisms and enzyme/protein functions, as well as the complicated cultivation of microorganisms [4]. The polyol method also had the limitations about the additives (e.g. hydroxyl ions, etc.) and the non-aqueous technique. In case of using polysaccharides (heparin, hyaluronic acid, chitosan, cellulose derivatives, starch, pectin, or alginate) as a reducing agent, metallic nanoparticles were successfully formed [18]. However, obstacles were reported as the complex purification and the poor solubility of polysaccharides in water. In contrast, plant extracts have been considered as a more favorable way, due to the biodiversity of plants, simple procedures, and the medicinal activities [11]. Especially, biosynthesized nanoparticles from plant extracts were more stable than that from microbes, and the reaction rate using plant extracts was more rapid [12]. Interestingly, the size and morphology through distinct parts/species or locations of plant species could be highly tunable. More importantly, plant extract-mediated methods may be used for a large-scale production [12].

Previously, diverse plants have been investigated regarding forming silver nanoparticles [19,20,21]. More than sixty plants used for green AgNP synthesis were summarized [11,14], but *Phyllanthus urinaria (P. urinaria)*, *Pouzolzia zeylanica (P. zeylanica),* and *Scoparia dulcis (S. dulcis)* are plants of interest that have not yet been explored. *P. urinaria, P. zeylanica,* and *S. dulcis* are traditional herbs distributed mainly in tropical countries [22,23,24,25,26,27]. In folk medicine, *P. urinaria* is well known as a remedy for treating many diseases, including diabetes, jaundice, malaria, cancer, infection, and liver diseases [24,25,26]. *P. zeylanica* shows its effects in anti-bacteria, anti-inflammation, anti-allergic, antioxidant, anti-snake venom activities, antiasthma, and pain relief [22,23,28]. *S. dulcis* is also known for its medicinal functions in treatment of inflammation, hemorrhoids, bronchitis, urinary disorders, diabetes, hypertension, kidney stones, stomach ailments, hepatosis, insect bites, fever, diarrhea, cancer, and ulcers [29,30].

In the study, *P. urinaria, P. zeylanica,* and *S. dulcis* were used as natural compounds to formulate silver nanoparticles. P.uri.AgNPs, P.zey.AgNPs, and S.dul.AgNPs were characterized by ultraviolet visible spectroscopy (UV-vis), the Fourier transform infrared spectroscopy (FTIR), and energy dispersive X-ray analysis (EDX) to confirm formation of nanoparticles, their functional groups, and elemental compositions. Scanning electron microscopy (SEM) and transmission electron microscopy (TEM) were performed to elucidate the nanoparticle morphology, the agglomeration and to calculate their dimension, especially to comprehend the difference between three types of these silver nanoparticles. Finally, *Aspergillus niger (A. niger), Fusarium oxysporum (F. oxysporum),* and *Aspergillus flavus (A. flavus)* were cultured on agar dishes containing P.uri.AgNPs, P.zey.AgNPs, and S.dul.AgNPs to investigate antifungal activities.

## 2. Materials and Methods 

### 2.1. Materials

Silver nitrate (AgNO_3_) was obtained from Guanhao High-Tech Co., Ltd. (Zhanjiang, China). Potassium bromide (KBr) were FT-IR grade and supplied by Sigma-Aldrich (Merck, Darmstadt, Germany). Potato dextrose agar (PDA) was purchased from Millipore (Merck, Kenilworth, NJ, USA). Deionized water (DIW) was obtained from Milli-Q HX 7150 systems (Merck Millipore, Alsace, France). pH-indicator paper was purchased from Merck (Darmstadt, Germany).

*P. urinaria, S. dulcis,* and *P. zeylanica* were planted in the medicinal garden of Tra Vinh University (Tra Vinh province, Vietnam). *A. niger, F. oxysporum,* and *A. flavus* were isolated and cultured by Institute of Applied Materials Science (Ho Chi Minh city, Vietnam). Deionized water (DIW) was used for all experiments.

**Figure 1:**
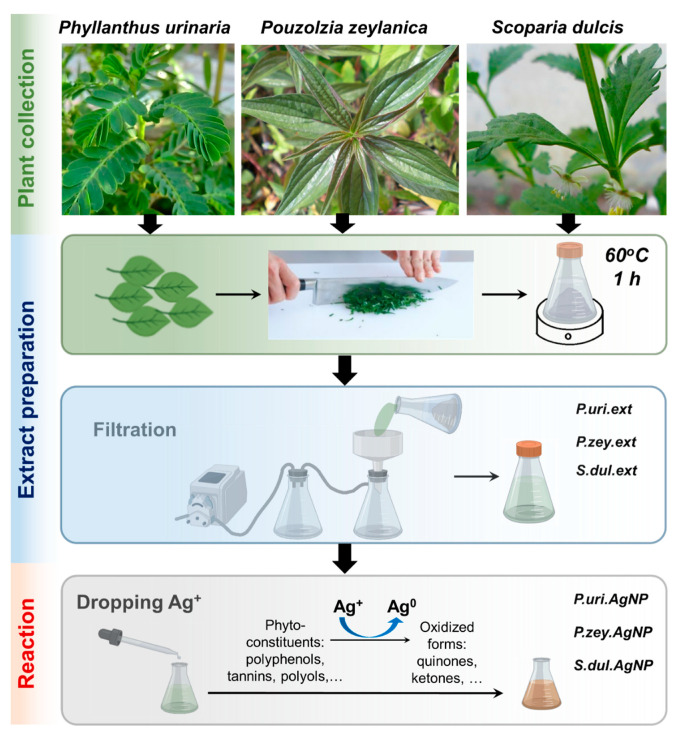
Biosynthesis procedure of silver nanoparticles using various plant extracts: *Phyllanthus urinaria*, *Pouzolzia zeylanica,* and *Scoparia dulcis* leaf extracts symbolized as P.uri.ext, P.zey.ext, and S.dul.ext were reacted with silver nitrate to form three types of silver nanoparticles that were named as P.uri.AgNP, P.zey.AgNP, and S.dul.AgNP, respectively.

Plant materials: *P. urinaria, P. zeylanica,* and *S. dulcis* leaves could not have been diseased, damaged, or contaminated. The leaves were collected and cut into small pieces. Each species (1 g) was put into the different Erlenmeyer flask and 50 mL of DIW was added. These Erlenmeyer flasks were heated at 60 °C for 60 min. Aqueous extracts of *P. urinaria, P. zeylanica,* and *S. dulcis* leaves (abbreviated as P.uri.ext, P.zey.ext and S.dul.ext respectively) were obtained after filtration with Whatman No. 1 filter paper (Figure 1). Their pH value was tested with pH-indicator papers. These extracts were kept at 4 °C for further usage within 7 days.

### 2.2. Preparation of Silver Nanoparticles Using P. urinaria, P. zeylanica, and S. dulcis Leaf Extracts

Silver nitrate of 1 mM was prepared in DIW. Three different Erlenmeyer flasks contained 8 mL of the P.uri.ext, P.zey.ext and S.dul.ext solutions. The silver nitrate solution (0.8 mL) was dropped slowly into each flask to form the silver nanoparticles at the rate of 30 drops per minute. Three silver nanoparticles fabricated by P.uri.ext, P.zey.ext, and S.dul.ext were named as P.uri.AgNPs, P.zey.AgNPs, and S.dul.AgNPs, respectively (Figure 1). The reactions were performed at room temperature. After 8 h, the reacted mixtures had yellowish-brown color, and were washed with DIW three times to collect silver nanoparticles.

### 2.3. Characterization of Silver Nanoparticles

*UV-Vis spectrophotometer:* To realize the formation of biosynthesized AgNPs, three silver nanoparticle solutions (P.uri.AgNPs, P.zey.AgNPs, and S.dul.AgNPs) and three leaf broths (P.uri.ext, P.zey.ext, and S.dul.ext) were respectively loaded into the quartz curvets to collect their UV-vis spectrum by the Shimadzu UV-1800 machine (Shimadzu, Columbia, MD, USA). The resolution was set at 1 nm, the wavelength range was 350–750 nm. DIW was used to adjust baseline.

*FTIR spectroscopy:* KBr was blended with each of all samples including the lyophilized P.uri.ext, P.zey.ext, S.dul.ext, P.uri.AgNPs, P.zey.AgNPs and S.dul.AgNPs in turn at the weight ratio of 100:1. These mixtures were pelleted and recorded by FTIR spectroscopy (Frontier MIR/FIR, PerkinElmer, Hopkinton, MA, USA). The wavenumber was set in the range of 500–4000 cm^−1^.

*EDX analysis:* The biosynthesized AgNPs including P.uri.AgNPs, P.zey.AgNPs, and S.dul.AgNPs in succession were dissolved in pure ethanol and followed by ultrasonication. A few microliters of these suspensions were loaded on copper grid, then dried in the air, and analyzed with EDX (H-7593, Horiba, Kyoto, Japan).

*SEM and TEM observation:* The sample preparation was similar to EDX measurement. The P.uri.AgNPs, P.zey.AgNPs, and S.dul.AgNPs were observed with TEM (JEOL-JEM-1400, JEOL Ltd., Tokyo, Japan) and SEM (S-4800, Hitachi, Tokyo, Japan).

### 2.4. Antifungal Assays

*A. niger, F. oxysporum,* and *A. flavus* were grown on the media containing various compositions to follow the fungal proliferation. The petri dishes containing pure PDA solution were used as the control (named as PDA). The petri dishes containing PDA and each of extracts including P.uri.ext, P.zey.ext, S.dul.ext were used to check the antifungal ability of each broth that was written in short as P.uri.ext, P.zey.ext, S.dul.ext. The petri dishes containing PDA and each biosynthesized AgNP at different concentrations were used to test the antifungal effect of these biosynthesized AgNPs. The symbolization was explained in Table 1.

After preparing all dishes, fungal colonies were placed directly in the center of agar plate. The fungal culture was carried out at room temperature. The diameter of fungal zone was measured every 24 h for 4 days.

### 2.5. Statistical Analysis

The results were replicated 3 times and represented as mean ± standard deviation. All experimental data were analyzed by Student’s t test. *P* < 0.05 implied that two compared results were statistically significant. *P* > 0.05 indicated non-statistical (NS) difference.

## 3. Results and Discussion

### 3.1. Biosynthesis of Silver Nnanoparticles from Plant Extracts

**Figure 2:**
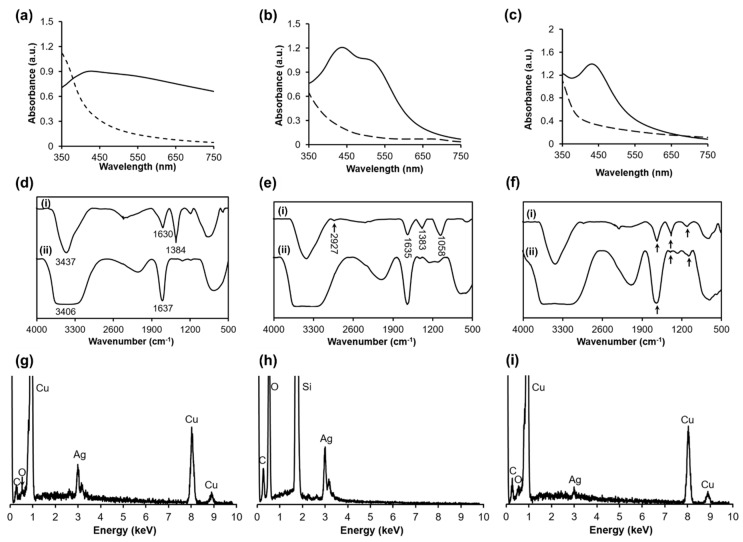
The UV-vis spectra of P.uri.ext and P.uri.AgNP (**a**), P.zey.ext and P.zey.AgNP (**b**), S.dul.ext and S.dul.AgNP (**c**), in which the dashed line is the spectra of extract, and the solid line is that of AgNP; the FTIR spectra of P.uri.ext and P.uri.AgNP (**d**), P.zey.ext and P.zey.AgNP (**e**), S.dul.ext and S.dul.AgNP (**f**), in which (i) line is the spectra of AgNP, (ii) line is the one of extract; the EDX spectra of P.uri.AgNP (**g**), P.zey.AgNP (**h**) and S.dul.AgNP (**i**).

Leaf extracts were obtained from three plant species including *P. urinaria, P. zeylanica,* and *S. dulcis* (Figure 1); and their pH value was around 7. Then, silver nitrate was simply added drop-by-drop to the extracts to form AgNPs under mild conditions (room temperature for 8 h). The mixture solution turned to yellowish brown to dark brown, demonstrating reactions between extracts and silver (I) ions. The UV-vis spectra of pure extracts (Figure 2a–c, dashed line) did not have any peaks in the wavelength range of 350–700 nm, and the peaks of spectra were at the wavelength around 200–280 nm, representing π − π* or n − π* transition of a myriad of organic compounds in plant extracts due to an absorbing wavelength of the UV radiation. The UV-vis of the reacted mixtures (Figure 2a–c, solid line) showed the peaks from 400–600 nm, demonstrating silver nanoparticles with a surface plasmon resonance (SPR) [31]. The nanoparticle size strongly impacted on the peak position: the larger scale had red-shifting (towards longer wavelengths), while the smaller one had blue-shifting (towards shorter wavelengths), because the conductive electrons of the aggregated nanoparticle surface become less flexible [31]. Concurrently, the pH value was reported as one of the influencing factors in the green synthesis of nanoparticles: a different pH displayed a different SPR behavior. At a neutral pH, the maximum wavelength was found in Figure 2a–c (dashed line) of three AgNPs that agreed with the previous study of Singh et al. At pH 7.5, green silver nanoparticles showed a peak of 449 nm [32].

The UV-Vis spectrum of P.uri.AgNPs (Figure 2a, solid line) had one broad peak at 400–550 nm. The P.zey.AgNP UV-vis spectrum (Figure 2b, solid line) exhibited two close peaks centered at 440 nm and 520 nm. The S.dul.AgNP one (Figure 2c, solid line) indicated the pointed peak of 430 nm. These UV-vis results predicted P.uri.AgNPs might agglomerate, the P.zey.AgNPs had widely size distribution, and the S.dul.AgNP dimension was smallest. This difference may be attributed by the phytoconstituent variance of each plant extracts. Table S1 summarizes the phytochemicals that indicate *P. urinaria, P. zeylanica,* and *S. dulcis* have different compounds; even various classes. *P. urinaria* possessed lignans, tannins, flavonoids, phenolics, terpenoids, and others. *P. zeylanica* had *nor-*lignans, carotenoids, vitamin C, malic acid, pectic acid, tartaric acid and gum beside tannins and flavonoids. *S. dulcis* mainly contained flavones, terpenes, and steroids. The underlying mechanism for the formation of silver nanoparticles in the plant extracts was an oxidation-reduction process. Generally, lignans, nor-lignans, tannins, flavonoids, phenolics, terpenoids, carotenoids, and vitamin C are great antioxidants and reducing agents [22,24,28,33]. These compounds, mostly possessing phenol or hydroxyl groups, may inhibit oxidants by transferring their hydrogen atom and forming a transition state pending one remained electron. In addition, the unsaturated chains of these phytoconstituents also may act as a reducer. The aldehyde functional groups of 5-hydroxymethyl-2-furaldehyde or p-hydroxybenzaldehyde may also react with oxidants to become carboxylic acids. *P. urinaria* contains the largest amount of antioxidants (Table S1) and quickly reduces Ag ions, forming relatively large nanoparticles.

Figure 2d–f(i) shows the FTIR spectra of P.uri.AgNPs, P.zey.AgNPs, and S.dul.AgNPs. These spectra indicated the presence of troughs at wavenumber region of 3500–3300 cm^−1^, 1630–1635 cm^−1^, 1380–1385 cm^−1^, and 1000–1100 cm^−1^, which were attributed to O–H stretching, O–H bending, C–H bending, C–O stretching vibration, respectively. These troughs were also found in the FTIR spectra of extracts (Figure 2d–f(ii)), but the OH troughs were broadening due to stronger hydrogen bonding. In contrast, P.uri.AgNPs, P.zey.AgNPs, and S.dul.AgNPs showed relatively sharp O–H stretching peaks, as compared to pure extracts, demonstrating that silver nanoparticles were capped by phytoconstituents [12,15]. So it could be said that using plant extracts could stabilize the formed silver nanoparticles within the synthesizing process. In fact, these biosynthesized silver nanoparticles including P.uri.AgNPs, P.zey.AgNPs, and S.dul.AgNPs had zeta potentials of −15.6 mV, −24.3 mV, and −20.8 mV, respectively (Figure S1). The negative potential implied that these green silver nanoparticles achieved a stable stage. The negative charge of P.uri.AgNPs, P.zey.AgNPs, and S.dul.AgNPs may explained by the carboxylic groups of phytochemicals from leaf extracts (Table S1). This value was also agreed with other AgNPs fabricated by phytochemicals [34]. In other words, the charge of AgNPs formed by gallic acid + quercetin, resveratrol, and mPEG-luteolin were −19.0 mV, −20.6 mV, and −25.5 mV respectively. The presence of carbon, oxygen, and silver was confirmed by EDX analysis (Figure 2g–i). The detected other elements such as Si and Cu were from the grid.

### 3.2. Size and Morphology of Biosynthesized Silver Nanoparticles (P.uri.AgNPs, P.zey.AgNPs and S.dul.AgNPs)

**Figure 3:**
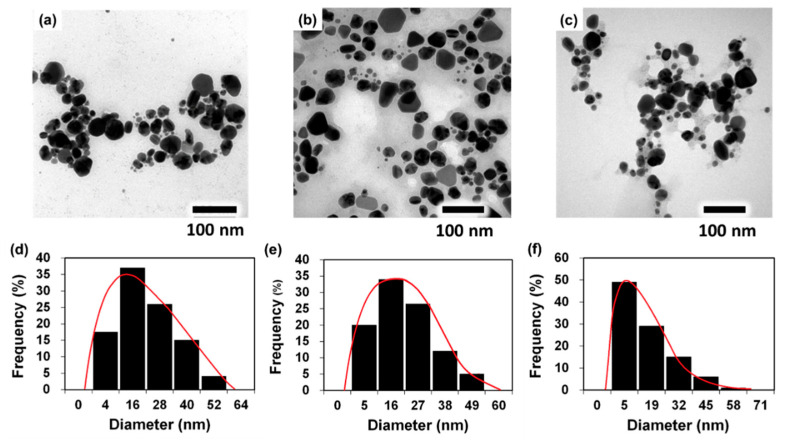
TEM images of P.uri.AgNP (**a**), P.zey.AgNP (**b**), and S.dul.AgNP (**c**); the graph of dimension distribution of P.uri.AgNP (**d**), P.zey.AgNP (**e**), and S.dul.AgNP (**f**).

Morphology and size are two of the most important properties of nanoparticles. SEM and TEM were used to look at P.uri.AgNPs, P.zey.AgNPs, and S.dul.AgNPs (Figure 3). The TEM images in Figure 3a reveals that P.uri.AgNPs were mostly spherical and oval. For the P.zey.AgNPs (Figure 3b), various morphologies such as spherical, triangle, plate-like polyshaped, pentagonal, and hexagonal shapes were found. S.dul.AgNPs were spherical and relatively smaller than P.uri.AgNPs and P.zey.AgNPs (Figure 3c), consistent with the results from UV-Vis (Figure 2c) and size measurements (Figure 3d–f). Also, their surfaces were smooth when observed by SEM (Figure S2). The size of P.uri.AgNPs, P.zey.AgNPs, and S.dul.AgNPs were in the range of 4–52 nm, 5–49 nm, and 5–45 nm. However, ~65% of P.uri.AgNPs had an average size of 28.3 nm and ~59% of P.zey.AgNPs was 26.7 nm, while ~50% of S.dul.AgNPs were of 5 nm. Taken together, the extract type impacted the size, morphology, and distribution of biosynthesized AgNPs. Considering influence factors, the reducing power [35] and capping agents [36] in synthesis process mainly decided nanoparticle structure. The strongly reducing compounds could form the small nanoparticles, while the weakly reducing agents led to making large and/or polydisperse nanoparticles [35]. However, the very rapid reduction may not give enough time for capping silver nanoparticles with phytoconstituents, thus may cause agglomeration. Hence, the S.dul.AgNPs were smallest due to the better harmony between the reducing power and capping reaction of S.dul.ext, compared to P.uri.ext and P.zey.ext broths. *P. urinaria* contains many well-known reductants such as rutin, kaempferol, quercetin, gallic acid, ellagic acid, 5-hydroxymethyl-2-furaldehyde, lignans, tannins, glycosides, rich of other polyols, and phenolics (Table S1) that show strong reducing activity. Similarly, the highly reducing content of *P. zeylanica* was exhibited by the presence of quercetin, epicatechin, ascorbic acid, gum, and alkaloids. Meanwhile, *S. dulcis* was found to contain fewer reducing compounds of phenolics, lignans, and diols than two aforementioned plants. Reasonably, S.dul.AgNPs had enough time to be grafted with its scoparic acid and others through –C=O groups [34] to be stabilized, thus became less aggregated than P.uri.AgNPs and P.zey.AgNPs. In addition, it may be understood that the size of P.uri.AgNPs and P.zey.AgNPs were approximately the same and similar to the other AgNPs formed by kaempferol, quercetin, or gallic acid reductant [34]. Three compounds were also found in *P. urinaria* and *P. zeylanica* (Table S1). Concerning the relation between nanoparticle morphology and capping agents, when increasing the content of capping agent, the diversified shapes such as rods, triangles, hexagon, cylinders and cubic would be formed besides the sphere. [36]. Thus it may be inferred that the P.zey.ext contained the highest capping compounds.

### 3.3. Antifungal Activities of P.uri.AgNPs, P.zey.AgNPs and S.dul.AgNPs

**Figure 4:**
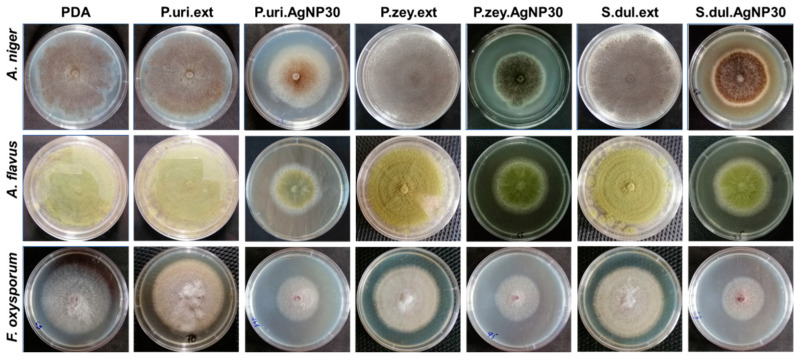
Three fungal strains including *A. niger, A. flavus,* and *F. oxysporum* were culture in different agar matrix after 96 h.

P.uri.AgNPs, P.zey.AgNPs, and S.dul.AgNPs were tested for antifungal ability against *A. niger, A. flavus,* and *F. oxysporum* at the concentration of 15, 30 and 45 ppm. The pure PDA and three extracts (P.uri.ext, P.zey.ext, and S.dul.ext) were parallelly carried out as a control. Figure 4 shows the growth of three fungal strains on various agar dishes for 96 h. The proliferation of *A. niger, A. flavus,* and *F. oxysporum* was suppressed by the presence of AgNPs in the nanoparticle concentration dependent manner, while the pure extracts were not influenced the proliferation of the fungus under the same concentration, which demonstrated the anti-fungal activities of P.uri.AgNPs, P.zey.AgNPs, and S.dul.AgNPs.

**Figure 5:**
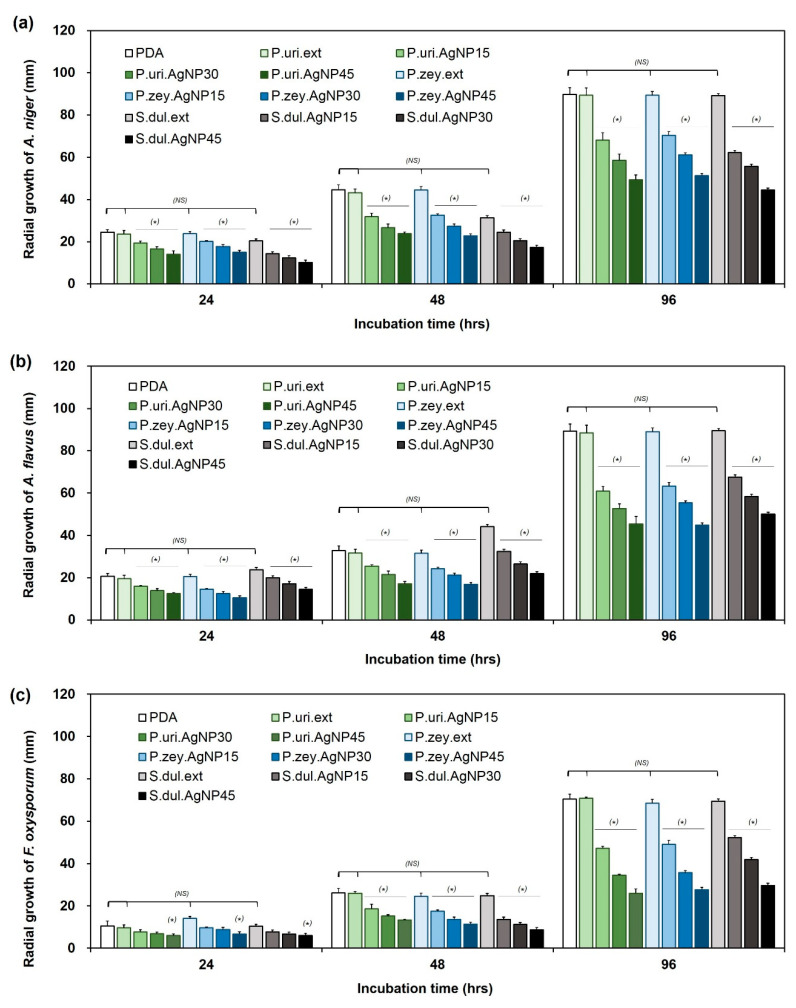
The mycelium diameter of *A. niger* (**a**), *A. flavus* (**b**)*,* and *F. oxysporum* (**c**) cultured on different agar matrices as a function of time interval. (PDA: potato dextrose agar dishes as a control; P.uri.ext, P.zey.ext, and S.dul.ext: the dishes made from PDA containing leaf extracts of *Phyllanthus urinaria, Pouzolzia zeylanica*, and *Scoparia dulcis*; P.uri.AgNP15, P.uri.AgNP30, and P.uri.AgNP45: the dishes made from PDA containing 15, 30 and 45 ppm of P.uri.AgNPs silver nanoparticles; P.zey.AgNP15, P.zey.AgNP30, P.zey.AgNP45: the dishes made from PDA containing 15, 30 and 45 ppm of P.zey.AgNPs silver nanoparticles; S.dul.AgNP15, S.dul.AgNP30, S.dul.AgNP45: the dishes made from PDA containing 15, 30 and 45 ppm of S.dul.AgNPs silver nanoparticles (*) *P* < 0.05; NS: non-statistical different.

Quantitatively, the mycelium diameters of *A. niger, A. flavus,* and *F. oxysporum* on various agar dishes were followed as a function of time interval until 96 h (Figure 5). The size of fungal zones was similar in P.uri.ext, P.zey.ext, and S.dul.ext, and gradually increased as incubated for a longer time period. There were no significant differences in the *A. niger* proliferation between PDA, P.uri.ext, P.zey.ext, and S.dul.ext dishes (Figure 5a). Similarly, *A. flavus* (Figure 5b)*,* and *F. oxysporum* (Figure 5c) showed the same trend of *A. niger*. In case of silver nanoparticles, the mycelium diameters became significantly smaller compared to PDA control. When increasing the biosynthesized AgNP concentration to 30 and 45 ppm, *A. niger* was increasingly inhibited by P.uri.AgNPs, P.zey.AgNPs, and S.dul.AgNPs. Taking an observation at Figure 5b,c shows the antifungal results against *A. flavus* and *F. oxysporum*, the similar comments with *A. niger* were withdrawn. So P.uri.AgNPs, P.zey.AgNPs, and S.dul.AgNPs may inhibit effectively all three fungal strains that was explained by their nanosize less than 60 nm. This dimension supports these nanoparticles to penetrate, to accumulate, and to interact with cell membranes easily, and thus inactivate the protein activities that leading cell death [37]. As a result, the AgNPs eco-friendly fabricated by the leaf broths of *P. urinaria, P. zeylanica,* and *S. dulcis* were proven their antifungal ability. P.uri.AgNPs, P.zey.AgNPs, and S.dul.AgNPs were less than 50 nm which size could exhibit an effective antimicrobial ability [14]. In previous studies, green AgNPs with size range less than 50 nm were also formed by other plants including *Elephantopus scaber, Phyllanthus amarus, Alpinia katsumadai, Psidium guajava, Salvia leriifolia,* and *Artocarpus altilis.* All showed the good antimicrobial activity against *A. flavus,* and *A. niger* besides *E. coli, Staphylococcus* spp., *Bacillus* spp., *Pseudomonas* spp. [14]. Higher antimicrobial activity was achieved when increasing green AgNP concentration [14]. So, together with previous ones, this study introduces three more renewable materials to produce silver nanoparticles as effective fungicide for biomedical and agricultural applications.

## 4. Conclusions

In the study, three kinds of silver nanoparticles (P.uri.AgNPs, P.zey.AgNPs, and S.dul.AgNPs) were successfully biosynthesized by simply adding silver (I) ions to herbal plant extracts *(P. urinaria, P. zeylanica, and S. dulcis)* under mild conditions. Their formation and physicochemical properties were well characterized by UV-Vis, FTIR, EDX, TEM, and SEM. The results revealed that P.uri.AgNPs, P.zey.AgNPs, and S.dul.AgNPs were modified with variously organic compounds in biosynthesis process. So the formation and the coating procedure of these silver nanoparticles were performed at the same time. The plant species possessing the different phytoconstituents that lead to influence on the size and morphology of silver nanoparticles. *P. urinaria* and *P. zeylanica* leaf extract could form the silver nanoparticles about 28.3 and 26.7 nm, especially *S. dulcis* leaf extract could create almost 5 nm particles. Among the three AgNP types, only P.zey.AgNP showed a diversified morphology including spherical, triangle, plate-like polyshaped, pentagonal, and hexagonal shapes, while the others were in spherical morphology. In addition, the antifungal ability of P.uri.AgNPs, P.zey.AgNPs, and S.dul.AgNPs against *A. niger, A. flavus,* and *F. oxysporum* were validated. This green method is the simplest and most largely scalable method for the production of antifungal silver nanoparticles in biomedical and agricultural applications.

## Figures and Tables

**Table 1:** The compositions of media prepared for antifungal assay. PDS = Potato dextrose agar,

Symbolization	Description
P.uri.AgNP15	15 ppm of P.uri.AgNPs in PDA
P.uri.AgNP30	30 ppm of P.uri.AgNPs in PDA
P.uri.AgNP45	45 ppm of P.uri.AgNPs in PDA
P.zey.AgNP15	15 ppm of P.zey.AgNPs in PDA
P.zey.AgNP30	30 ppm of P.zey.AgNPs in PDA
P.zey.AgNP45	45 ppm of P.zey.AgNPs in PDA
S.dul.AgNP15	15 ppm of S.dul.AgNPs in PDA
S.dul.AgNP30	30 ppm of S.dul.AgNPs in PDA
S.dul.AgNP45	45 ppm of S.dul.AgNPs in PDA

## Supplementary Materials

The following are available online at https://www.mdpi.com/2079-4991/10/3/542/s1, Figure S1: The zeta potential of P.uri.AgNP (a), P.zey.AgNP (b) and S.dul.AgNP (c); Figure S2: The SEM images of P.uri.AgNP (a), P.zey.AgNP (b) and S.dul.AgNP (c); Table S1: The summary of phytoconstituents in *P. urinaria, P. zeylanica*, and *S. dulcis*.
