# Correction: Nguyen et al. Green Silver Nanoparticles Formed by *Phyllanthus urinaria*, *Pouzolzia zeylanica*, and *Scoparia dulcis* Leaf Extracts and the Antifungal Activity. *Nanomaterials* 2020, *10*, 542

**DOI:** 10.3390/nano14050475

**Published:** 2024-03-06

**Authors:** Dai Hai Nguyen, Jung Seok Lee, Ki Dong Park, Yern Chee Ching, Xuan Thi Nguyen, V.H. Giang Phan, Thai Thanh Hoang Thi

**Affiliations:** 1 Institute of Applied Materials Science, Vietnam Academy of Science and Technology, 01 TL29 District 12, Ho Chi Minh City 700000, Vietnam; nguyendaihai0511@gmail.com; 2 Graduate University of Science and Technology, Vietnam Academy of Science and Technology, Hanoi 100000, Vietnam; 3 Biomedical Engineering, Malone Engineering Center 402A, Yale University, 55 Prospect St., New Haven, CT 06511, USA; jungseok.lee@yale.edu; 4 Department of Molecular Science and Technology, Ajou University, Suwon 16499, Republic of Korea; kdp@ajou.ac.kr; 5 Department of Chemical Engineering, Faculty of Engineering, University of Malaya, Kuala Lumpur 50603, Malaysia; chingyc@um.edu.my; 6 Biomaterials and Nanotechnology Research Group, Faculty of Applied Sciences, Ton Duc Thang University, Ho Chi Minh City 700000, Vietnam; 186002007@student.tdtu.edu.vn

## Error in Figure

In the original publication [1], there was a mistake in Figure 4 as published. An error occurred when we arranged the images, resulting in the display of two identical images. The corrected Figure 4 appears below. The authors state that the scientific conclusions are unaffected. This correction was approved by the Academic Editor. The original publication has also been updated.

## Figures and Tables

**Figure 4:**
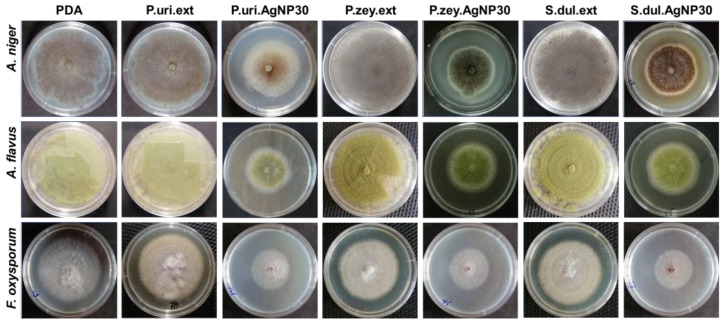
Three fungal strains including *A. niger*, *A. flavus*, and *F. oxysporum* were culture in different agar matrix after 96 h.
